# Discrimination of Stem Cell Status after Subjecting Cynomolgus Monkey Pluripotent Stem Cells to Naïve Conversion

**DOI:** 10.1038/srep45285

**Published:** 2017-03-28

**Authors:** Arata Honda, Yoshihiro Kawano, Haruna Izu, Narantsog Choijookhuu, Kimiko Honsho, Tomonori Nakamura, Yukihiro Yabuta, Takuya Yamamoto, Yasuhiro Takashima, Michiko Hirose, Tadashi Sankai, Yoshitaka Hishikawa, Atsuo Ogura, Mitinori Saitou

**Affiliations:** 1 Organization for Promotion of Tenure Track, University of Miyazaki, 5200, Kibara, Kiyotake, Miyazaki 889-1692, Japan; 2 RIKEN BioResource Center, Tsukuba, Ibaraki 305-0074, Japan; 3 Department of Anatomy, Histochemistry and Cell Biology, Faculty of Medicine, University of Miyazaki, Miyazaki 889-1692, Japan; 4 Department of Anatomy and Cell Biology, Graduate School of Medicine, Kyoto University, Yoshida-Konoe-cho, Sakyo-ku, Kyoto 606-8501, Japan; 5 JST, ERATO, Yoshida-Konoe-cho, Sakyo-ku, Kyoto 606-8501, Japan; 6 Center for iPS Cell Research and Application, Kyoto University, 53 Kawahara-cho, Shogoin, Sakyo-ku, Kyoto 606-8507, Japan; 7 Institute for Integrated Cell-Material Sciences, Kyoto University, Yoshida-Ushinomiya-cho, Sakyo-ku, Kyoto 606-8501, Japan; 8 AMED-CREST, AMED, 1-7-1 Otemachi, Chiyoda-ku, Tokyo 100-0004, Japan; 9 Tsukuba Primate Research Center, National Institute of Biomedical Innovation, Health and Nutrition, Tsukuba, Ibaraki 305-0843, Japan

## Abstract

Experimental animal models have played an indispensable role in the development of human induced pluripotent stem cell (iPSC) research. The derivation of high-quality (so-called “true naïve state”) iPSCs of non-human primates enhances their application and safety for human regenerative medicine. Although several attempts have been made to convert human and non-human primate PSCs into a truly naïve state, it is unclear which evaluation methods can discriminate them as being truly naïve. Here we attempted to derive naïve cynomolgus monkey (Cm) (*Macaca fascicularis*) embryonic stem cells (ESCs) and iPSCs. Several characteristics of naïve Cm ESCs including colony morphology, appearance of naïve-related mRNAs and proteins, leukaemia inhibitory factor dependency, and mitochondrial respiration were confirmed. Next, we generated Cm iPSCs and converted them to a naïve state. Transcriptomic comparison of PSCs with early Cm embryos elucidated the partial achievement (termed naïve-like) of their conversion. When these were subjected to *in vitro* neural differentiation, enhanced differentiating capacities were observed after naïve-like conversion, but some lines exhibited heterogeneity. The difficulty of achieving contribution to chimeric mouse embryos was also demonstrated. These results suggest that Cm PSCs could ameliorate their *in vitro* neural differentiation potential even though they could not display true naïve characteristics.

Embryonic stem cells (ESCs) and induced pluripotent stem cells (iPSCs) have been evaluated for several characteristics to confirm their pluripotency. Improvements in quality and evaluations of their stemness are important for developing medical applications and chimeric animal production. The PSC state can be distinguished by two terminological definitions: naïve and primed^1^. The naïve pluripotent state remains similar to that of the inner cell mass from mouse pre-implantation blastocysts^2^, and it can be induced by the generation of mouse ESCs or reprogramming of mouse somatic cells to form iPSCs^3^. On the other hand, the primed pluripotent state can be defined as having the characteristics of epiblast stem cells (EpiSCs), which are generated from mouse 5.5–7.5 days post coitus (dpc) post-implantation epiblasts^4^,^5^ or epiblast like cells (EpiLCs), which are similar to pre-gastrulating epiblasts^6^,^7^. Although naïve PSCs can effectively contribute to chimeras when introduced into pre-implantation embryos and can differentiate into germ cells *in vivo* and *in vitro*, primed PSCs show a restricted capacity for this behaviour^8^,^9^,^10^. Other than rodents, almost all mammals including human-derived PSCs show a primed pluripotent state. The successful conversion of primed-state PSCs into a naïve state might enable the derivation of high-quality human PSCs.

Primed PSCs from several animal species have been attempted to convert into a naïve state^11^,^12^,^13^. Properties of a naïve state are a domed-shape colony morphology, leukaemia inhibitory factor (LIF) dependency, ability to use mitochondrial respiration, pluripotent transcriptomal circuitry, transcriptional status resembling pre-implantation epiblasts, and efficient ability to contribute to chimeric embryos with subsequent germline transmission. However, such PSCs failed to fulfil these conditions (especially showing transcriptomal similarity to pre-implantation epiblasts); therefore, they should be termed not “naïve”, but “naïve-like”. These exhibit several naïve-specific characteristics but fail to acquire a transcriptome status resembling pre-implantation epiblasts and can apparently be distinguished from primed-state PSCs (see Supplementary Table S1). However, many types of PSCs in several mammalian species, which researchers had hoped to convert into a true naïve state, could not fulfil these naïve properties. For humans, it is difficult to evaluate the ability of PSCs to contribute to chimeras because of technical and ethical concerns, so evaluating gene transcriptional networks before and after naïve conversion has been achieved is recognized as one of the most precise means of checking on “true” naïve pluripotency^14^. Actually, the overexpression of *KLF2* and *NANOG* when cultured in appropriate conditions provided primed-state human ESCs with putative naïve pluripotency, and these showed significant overlap with mouse ESCs and human pre-implantation embryos in terms of their transcriptome status^15^,^16^. To mitigate the ethical concerns in using human PSCs, translational research using non-human primates might provide precise indicators for human PSC application. Naïve-like conversion of non-human primate ESCs^17^ and iPSCs^18^ has been demonstrated. Both types of naïve-like converted PSCs could contribute to chimeric embryos but only in minor ways via unknown species-specific features despite the successful demonstration of some naïve characteristics. To develop efficient human translational research, it will be necessary to evaluate and compare detailed characteristics before and after achieving naïve conversion of ESCs and iPSCs in non-human primates.

In this report, we have attempted to generate naïve ESCs and iPSCs from cynomolgus monkey (Cm) (*Macaca fascicularis*) cells by subjecting them to a naïve conversion system that we developed for rabbit ESCs with some modifications. Transcriptome evaluations with pre- and post-implantation Cm embryos^7^ clearly revealed the precise status of the PSCs: not naïve but naïve-like. However, they showed enhanced potential for neural differentiation *in vitro* after conversion. This study offers valuable pointers for deriving true naïve PSCs while avoiding irrelevant or misleading evidence of naïve-like features.

## Results

### Subjecting Cm ESCs to modified rabbit naïve conversion

**Figure 1:**
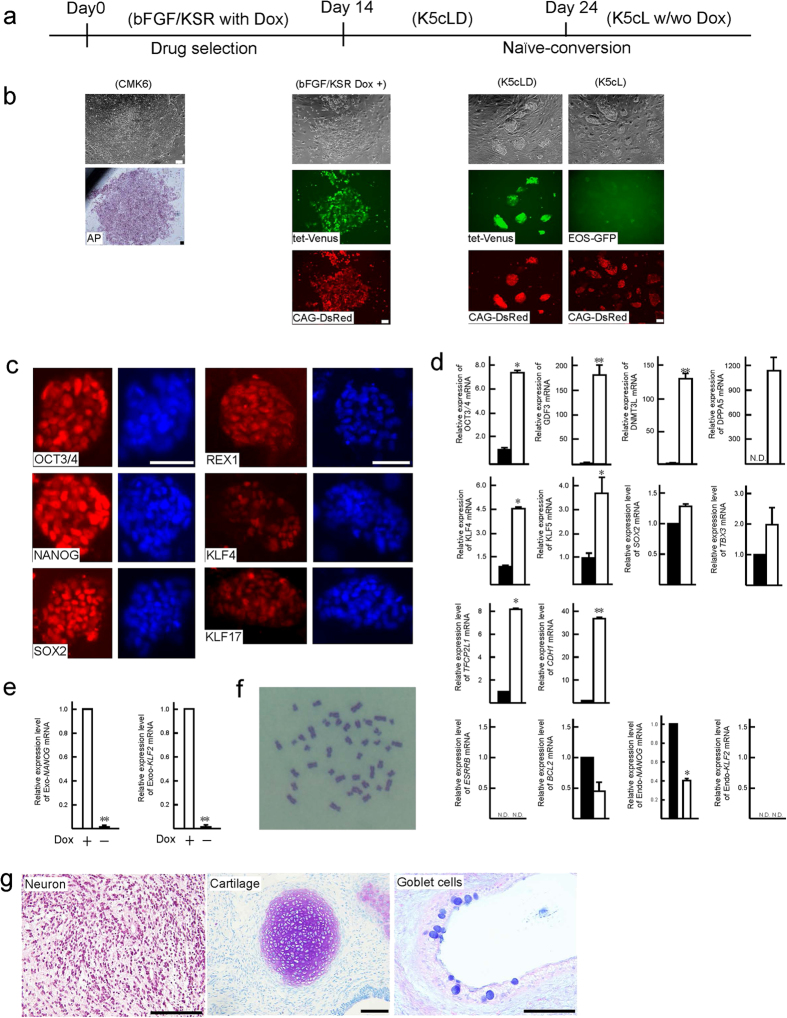
Naïve-like conversion of cynomolgus monkey (Cm) ESCs. (**a**,**b**) Schematic representation of time schedule (**a**) and representative images (**b**) of the naïve-like conversion of a Cm ESC line, CMK6, observed at different stages during naïve-like conversion. AP, alkaline phosphatase activity. Scale bar = 100 μm. (**c**) Immunocytochemical analysis of the CMK6 cell line that had been converted into a naïve-like state, using antibodies (OCT3/4, NANOG, SOX2, REX1, KLF4, and KLF17) directed against markers of naïve pluripotency-related proteins. Scale bar = 50 μm. (**d**) RT–qPCR for naïve pluripotency-related transcripts before (closed bars: primed) and after (open bars: naïve-like) naïve-like conversion. Error bars indicate the standard deviation (S.D.) *P < 0.05; **P < 0.01. (**e**) RT–qPCR confirmation of the expression and silencing of Exo-*hNANOG* and Exo-*hKLF2* in the presence (+) or in the absence (−) of Dox. (**f**) Normal number (2n = 42) of metaphase chromosomes confirmed in naïve-like converted CMK6 cells at passage 12. (**g**) Teratoma formation by naïve-like converted CMK6. Various tissues of the three germ layer origins are identified: neurons stained with haematoxylin and eosin (ectoderm), hyaline cartilage stained with Toluidine blue (mesoderm), and goblet cells stained with Alcian blue (endoderm). Scale bar = 100 μm.

A Cm ESC line, CMK6, was first subjected to methods for naïve conversion using a system that had been developed for rabbit PSCs^12^,^13^ with some modifications. This protocol uses overexpression of the *OCT3/4* gene by the EF1α promoter in rabbit PSCs, which are cultured in a medium containing knock-out serum replacement (KSR), and three chemical inhibitors: CHIR99021, a glycogen synthase kinase (GSK)-3β inhibitor; forskolin, an adenylyl cyclase activator; kenpaullone, a GSK-3β and cyclin-dependent kinase inhibitor; as well as human LIF. This medium is termed K3cL. Kenpaullone is known to express strong reprogramming activity, not only as a GSK-3β or cell cycle inhibitor, but it also has uncharacterized desirable effects^19^. When a Cm ESC line, CMK6, was cultured in K3cL medium without transgenic overexpression of *OCT3/4*—as used for rabbit PSC naïve conversion—almost all the colonies differentiated and disappeared within five passages. This result indicated the requirement of exogenous trigger(s) to maintain pluripotency at an initial phase of naïve conversion. To control and monitor such exogenous triggers, the plasmid vectors PB-tet-*hKLF2*-*Venus* and PB-tet-*hNANOG-Venus* were transfected into CMK6 cells and cultured with doxycycline (Dox) as reported by Takashima *et al*.^15^ and Theunissen *et al*.^16^. PB-EOS-*GFP*-*puroR*^20^ and PB-CAG-*Su9DsRediN* were also transfected to confirm pluripotency and the ability to contribute to chimeric offspring, respectively. The EOS-containing vector also has a reverse tetracycline transactivator (rtTA) cassette for enhanced expression of the tet promoter^21^. Two weeks after transfection and drug selection using primed culture medium containing basic fibroblast growth factor (bFGF) and KSR in the presence of Dox, almost all the colonies showed Venus and DsRed signals (Fig. 1b). When a CMK6 line overexpressing *KLF2* and *NANOG* was cultured in *t* (titrated) 2i (CHIR99021 and a MEK inhibitor, PD0325901) with LIF medium in the presence of Dox (*t*2iLD) according to Takashima *et al*.^15^, Cm ESCs could not proliferate in the same way as human naïve ESCs, and some differentiated colonies appeared (see Supplementary Fig. S1). This result suggests that Cm ESCs could not be maintained by these conditions even when exogenous *KLF2* and *NANOG* were overexpressed, as seen with human naïve conversion. However, after transfection with *KLF2* and *NANOG* as exogenous triggers, transgenic CMK6 cells could be maintained in K3cL medium with Dox (K3cLD) with the appearance of some of differentiated (cobble-stone-like) colonies (see Supplementary Fig. S1). To prevent the appearance of differentiated colonies, a BRAF inhibitor, SB590885, and a PKC inhibitor, Gö6983, which are known to be effective for deriving naïve human PSCs in conditions overexpressing *KLF2* and *NANOG*, were added to K3cLD (now termed K5cLD medium). When this K5cLD medium was used, colonies become tightly packed and dome-shaped, as seen in mouse ESCs (Fig. 1b). These cells could be propagated by single cell dissociation using trypsin digestion even in the absence of a rho-associated protein kinase inhibitor. In the presence of Dox, a bright Venus signal that was tandemly expressed with exogenous *hKLF2* and *NANOG* appeared, and undifferentiated colonies could be maintained for over half a year. At 24 days after conversion by Dox treatment, domed colonies could be maintained without Dox. In the absence of Dox, faint green fluorescent protein (GFP) signals derived from the EOS-*GFP* vector appeared, as seen in human PSCs^15^ (Fig. 1b). However, some differentiated colonies appeared and gradually increased after Dox withdrawal (see Supplementary Fig. S2). To maintain their undifferentiated status without any differentiated cells, culture medium supplemented with Dox (K5cLD) was used. Exogenous *KLF2* and *NANOG* were both expressed in the presence of Dox and silenced in its absence (Fig. 1e). We have provisionally termed this domed-shaped stem cell status to be “naïve-like”. These naïve-like CMK6 cells were immunochemically positive for naïve pluripotency-related marker proteins (Fig. 1c). Naïve-like CMK6 cells were strongly positive for OCT3/4, NANOG, and SOX2, but weakly positive for REX1, KLF4, and KLF17. However, some heterogeneity could be observed in KLF4 expression. The increased expression of *OCT3/4, GDF3, DNMT3L, DPPA5, SOX2, TBX3, TFCP2L1, CDH1, KLF4*, and *KLF5*, which are candidate genes for maintaining naïve pluripotency, was confirmed by quantitative reverse transcription polymerase chain reaction (RT–qPCR; Fig. 1d). However, unchanged or reduced expressions were confirmed in endo-*KLF2*, endo-*NANOG, BCL2*, and *ESRRB*. The addition of Dox to prevent the appearance of differentiated colonies could substitute for Endo-*KLF2* and Endo-*NANOG* and potentially compensate for naïve-related transcriptional circuitry (Fig. S2). Normal karyotypes (Fig. 1f and Supplementary Table S2) and typical teratoma-forming activity (Fig. 1g and Supplementary Fig. S3) were verified after 10 passages in the naïve culture conditions. As reported by Takashima *et al*.^15^, naïve-like CMK6 cells reverted to a conventional flat ESC colony morphology by changing the K5cLD medium to the primed culture medium (bFGF/KSR) (see Supplementary Fig. S4). These observations imply that Cm ESCs were successfully converted into a naïve-like state without losing their pluripotency.

### Effectiveness of LIF and Dox for maintaining their pluripotency in Cm ESCs

**Figure 2:**
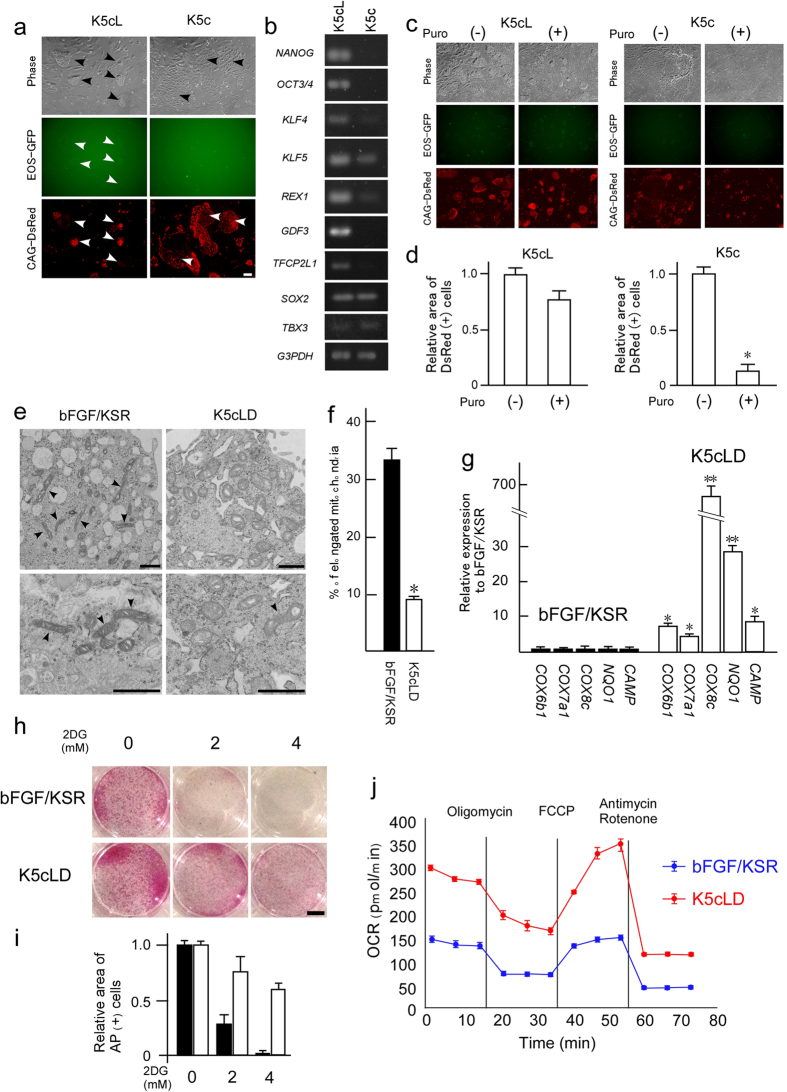
Reactivity of LIF and mitochondrial alterations according to naïve-like conversion of Cm ESCs. (**a**) Phase contrast images (Phase), EOS-GFP, and CAG-Su9DsRed signals of naïve-like Cm ESCs cultured in the presence (K5cL medium) and the absence (K5c) of LIF. After withdrawal of LIF, cobble-stone-like colonies appeared, and faint EOS-GFP signals disappeared completely. Scale bar = 100 μm. (**b**) Semi-quantitative RT–PCR of naïve-like Cm ESCs before and after withdrawal of LIF. The mRNA expression of all examined naïve pluripotency-related genes decreased or disappeared after the withdrawal of LIF. (**c** and **d**) Puromycin resistance assay using naïve-like Cm ESCs before and after the withdrawal of LIF. Colony morphology and fluorescence signals are shown as images (**c**) and as quantitative evaluations (**d**). In the absence of LIF (–), almost all naïve-like Cm ESCs disappeared with puromycin treatment. (**e** and **f**) Electron microscopic images (**e**) and their quantification (**f**) of the mitochondria in Cm ESCs before (bFGF/KSR) and after (K5cLD) naïve-like conversion. Before naïve-like conversion, primed-state ESCs contained more elongated mitochondria (arrowheads) than naïve-like converted cells. Scale bar = 1 μm. (**g**) RT–qPCR of potential regulators involved in mitochondrial respiration, *COX6b1, COX7a1, COX8c, NQO1*, and cytochrome oxidase (COX)-associated mitochondrial protein (*CAMP*) before (bFGF/KSR medium, closed bars) and after (K5cLD medium, open bars) naïve-like conversion. All genes examined were upregulated by naïve-like conversion. (**h** and **i**) AP positive colony formation (**h**) and quantification (**i**) with 2-deoxyglucose (2DG) treatment. Scale bar = 1 cm. Within a week, almost all Cm ESCs in primed culture conditions (bFGF/KSR) disappeared in the presence of 4 mM of 2DG. (**j**) Graph of OCR changes in naïve-like ESCs and primed ESCs. Error bars indicate the S.D. *P < 0.05; **P < 0.01.

Naïve-like converted Cm ESCs could be maintained in the absence of bFGF. If these cells had a true naïve state, strict sensitivity to LIF should have been shown^22^. Additionally, we needed to address the effects of Dox on their pluripotency. To test these effects, LIF and Dox were removed from K5cLD medium. Seven days after LIF and Dox removal (K5c), the colony morphology changed into a cobble-stone appearance, and the faint EOS-GFP signals disappeared completely (Fig. 2a). Semi-quantitative RT–PCR demonstrated that the expression of pluripotency-linked genes disappeared or was decreased by this treatment without *SOX2* or *TBX3* (Fig. 2b). To evaluate the effect of LIF on naïve-like status, the EOS vector, known as an indicator of pluripotency, was applied. Because the EOS vector drives the expression of *GFP* and *puroR* genes via their EOS promoter, naïve-like Cm ESCs were cultured with puromycin in the presence or absence of LIF. In spite of the absence of Dox (K5cL medium), more than 80% of cells survived; however, almost all of the naïve-like Cm ESCs died within a week after the withdrawal of LIF (Fig. 2c,d). These results suggest that LIF and Dox together acted in the maintenance of pluripotency in Cm ESCs and also demonstrated that removing both of them from the cultures induced rapid loss of their undifferentiated status.

### Acquisition of mitochondrial respiration by naïve-like conversion

Human naïve and primed ESCs exhibit distinct metabolic profiles, and they convert markedly, with changes in mitochondrial respiration and glycolysis^23^,^24^. It is known that this primed-to-naïve switch leads from an exclusively glycolytic metabolism in the primed state to both glycolytic and mitochondrial respiration in the naïve state. To examine the functional consequences of such altered metabolic properties, several characteristics were evaluated. Electron microscopy before and after naïve-like conversion showed that in the primed state, the Cm ESCs had many elongated mitochondria with a dense matrix, in contrast to the mitochondria of naïve-like converted Cm ESCs, which are round to oval, displaying sparse and irregular cristae and an electron-lucent matrix (Fig. 2e,f). RT–qPCR demonstrated that the mRNA expression levels of mitochondrial function-related genes were increased significantly by naïve-like conversion of Cm ESCs (Fig. 2g). When an inhibitor of glycolysis, 2-deoxyglucose (2DG), was added, the primed-state Cm ESCs disappeared because of their exclusive dependency on glycolytic metabolism (Fig. 2h,i). On the other hand, naïve-like converted Cm ESCs persisted and formed alkaline phosphatase (AP)-positive colonies due to the utilization of acquired mitochondrial respiration. Moreover, Cm CMK6 cells were examined a basal oxygen consumption rate (OCR) and we found that it was substantially higher in naïve-like CMK6 than in primed-state cells (Fig. 2j). These results suggest that the primed-state Cm ESCs acquired the capacity for mitochondrial respiration by naïve-like conversion.

### Naïve-like conversion of Cm iPSCs

**Figure 3:**
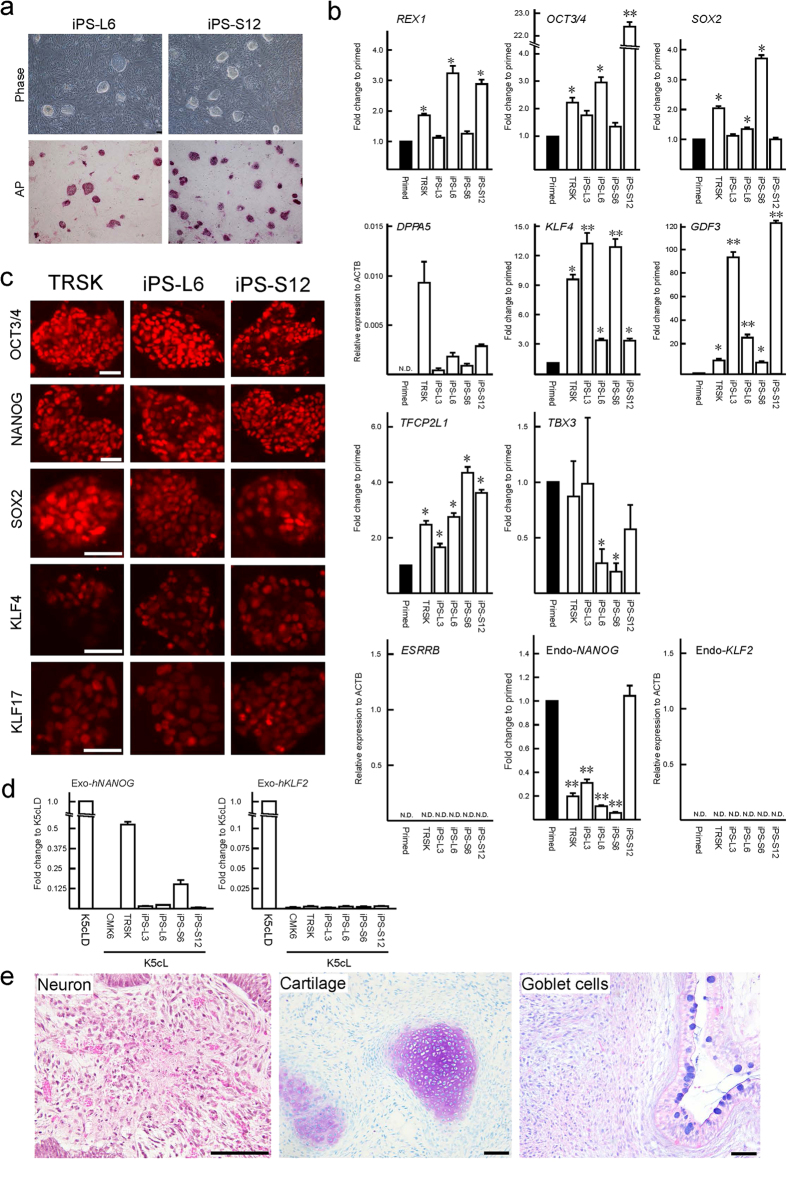
Naïve-like conversion of Cm iPSC lines. (**a**) Phase contrast images (Phase) and the alkaline phosphatase activity (AP) signals of naïve-like Cm iPSCs. (**b**) RT–qPCR for naïve pluripotency-related transcripts before (closed bars: primed) and after (open bars: naïve-like) naïve-like conversion of a Cm ESC line, TRSK, and iPSC lines. Expression levels before (primed) and after naïve-like conversion of each cell line are standardized to 1.0. *DPPA5* and endo*-KLF2* indicate the relative expression level of *ACTB* (α-actin). Error bars indicate the S.D. *P < 0.05; **P < 0.01. (**c**) Immunocytochemical analysis of PSC lines (TRSK, iPS-L6, and iPS-S12) that had converted into a naïve-like state, using antibodies directed against markers of naïve pluripotency related proteins. (**d**) Relative expression of Exo-*hNANOG* and Exo-*hKLF2* in the presence (K5cLD medium) and in the absence (K5cL medium) of Dox. (**e**) Teratoma formation of a naïve-like converted iPSC line, iPS-S12. Various tissues of the three germ layers are identified: neurons stained with HE (ectoderm), hyaline cartilage stained with Toluidine blue (mesoderm), and goblet cells stained with Alcian blue (endoderm). Scale bar = 100 μm.

To compare ESCs and iPSCs before and after naïve-like conversion, Cm iPSC lines were derived from freshly collected liver and stomach of a female Cm foetus (see Supplementary Fig. S5). Episomal plasmid vectors bearing *OCT3/4, SOX2, KLF4, LIN28, L-MYC*, and *p53* small hairpin RNA were transduced into cultured somatic cells, and then iPSC generation was carried out. The frequency of derivation of iPSC lines was determined from the liver (termed iPS-L) giving 12 lines (0.024%), and the stomach (termed iPS-S) giving 16 lines (0.170%). Cm iPSCs could be maintained by primed culture conditions as Cm ESCs (see Supplementary Fig. S5). Alkaline phosphatase activity and the expression of pluripotency-related genes were confirmed (see Supplementary Fig. S5). Generally, the episomal vectors disappeared spontaneously during this process. However, iPS-L3 and iPS-S6 cell lines showed persistent expression of exogenous *OCT3/4* (see Supplementary Fig. S5). Actually, an episomal vector, pCXLE-hOCT3/4-shp53-F, was detected in iPS-L3, iPS-L6, and iPS-S6 cell lines by genomic PCR (see Supplementary Fig. S5). Generated iPSC lines showed a normal karyotype (2*n *=* *42) at passages 10–20 (see Supplementary Table S1). When these iPSC lines were transplanted into severe combined immunodeficient mice, typical teratomas with all three primary germ layers were generated within 2 months after transplantation from all iPSC lines examined (see Supplementary Fig. S5 and Table S3). Liver-derived iPSC lines (L3 and L6) and stomach-derived iPSC lines (S6 and S12) were subjected to naïve-like conversion as ESCs. The colony morphology of naïve-like converted Cm iPSCs was dome shaped and positive for AP activity and naïve pluripotency-related proteins, as with naïve-like ESCs (Fig. 3a,c). Increased mRNA expressions of *REX1, OCT3/4, GDF3, DPPA5, SOX2, TFCP2L1*, and *KLF4* were observed after naïve-like conversion of iPSCs (Fig. 3b). As with naïve-like ESCs, expression of endo-*KLF2*, endo-*NANOG*, and *ESRRB* did not increase in all converted iPS cell lines (Fig. 3b). However, unlike naïve-like CMK6 cells, the naïve pluripotency-related marker *TBX3* did not increase with conversion. Exogenous h*NANOG* and h*KLF2* were silenced in almost all iPSC lines examined (Fig. 3d). However, some remaining expression of Exo-h*NANOG* was detected in a naïve-like converted ESC line, TRSK, and an iPSC line, iPS-S6. Teratomas able to form all three primary germ layers were also confirmed in all naïve-like converted iPSC lines (Fig. 3e, Supplementary Fig. S6, and Supplementary Table S3).

### Transcriptomal analysis of Cm ESCs and iPSCs before and after naïve-like conversion

**Figure 4:**
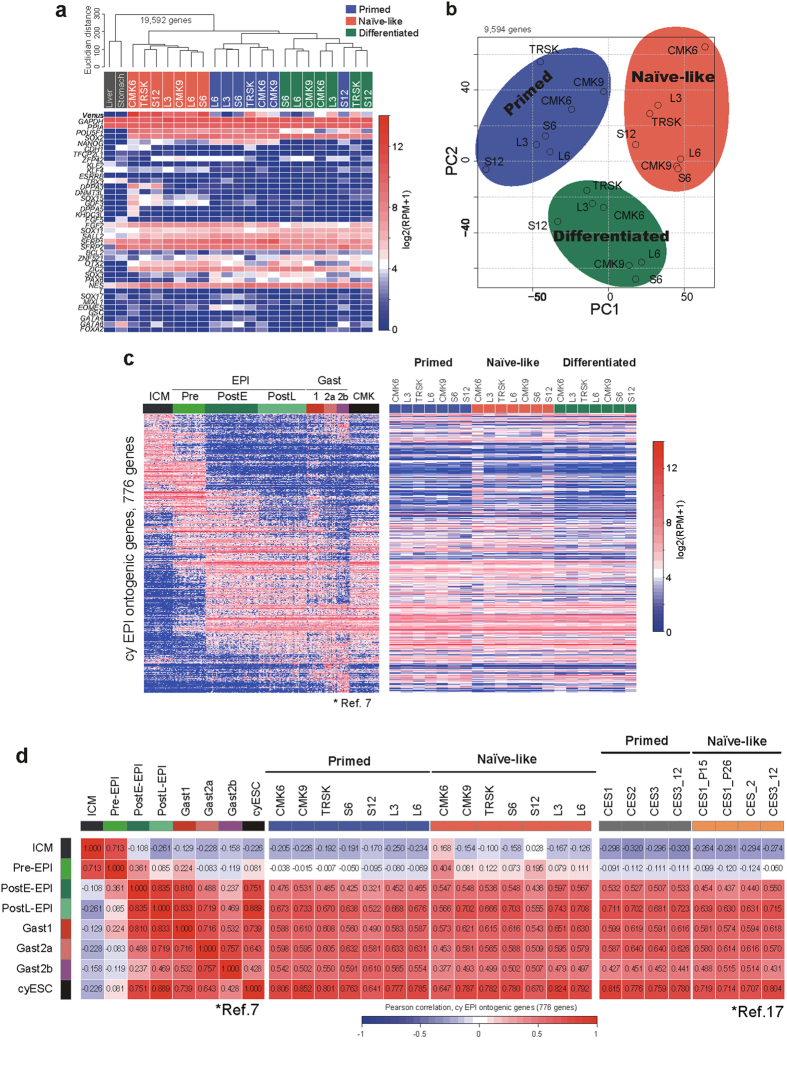
Transcriptome evaluation of naïve-like Cm PSCs by SC3-seq. (**a**) UHC of Cm somatic cells and PSCs cultured in bFGF/KSR (primed medium), K5cLD (naïve-like medium), and K5c (differentiated medium) by all expressed genes (log_2_(RPM + 1) > 4 in at least one sample among PSC lines; 19,562 genes) and heat maps of the levels of selected marker genes. Colour bars under the dendrogram indicate the cell types (grey, somatic cells; red, naïve-like PSCs; blue, primed PSCs; green, differentiated). (**b**) PCA of PSCs cultured in bFGF/KSR (blue: primed), K5cLD (red: naïve-like), and K5c (green: differentiated). (**c**) Heat map of the expression of 776 Cm EPI ontogenic genes in Cm embryos (ICM, Epiblast (Pre-, PostE-, and PostL-), Gastrulating (1, 2a, and 2b) and Cm ESCs (CMK6, TRSK, and CMK9) and Cm iPSCs cultured in bFGF/KSR (primed medium), K5cLD (naïve-like medium), and K5c (differentiated medium). (**d**) Heat map of the correlation coefficients among embryonic cells (Cm embryo), PSCs (primed, naïve-like), and those reported by refs 7 and 17. Correlation coefficients were calculated using the averaged expression levels of genes in (**c**).

To ascertain whether naïve-like conversion was achieved successfully and to what degree it was accomplished, the transcriptome status of Cm PSCs cultured in bFGF/KSR (primed) medium, K5cLD (naïve-like) medium, and K5c medium (differentiated) was compared with that known for Cm embryos^7^. Unsupervised hierarchical clustering (UHC) of Cm PSCs and the expression patterns of key genes are shown by a heat map (Fig. 4a). Principal component analysis (PCA) with all expressed genes in Cm PSCs was also done (Fig. 4b). These transcriptome evaluations suggested that PSCs in primed, naïve-like, and differentiated states could be classified according to their culture conditions. Although variation was observed in naïve-like PSCs, the transcriptome status of CMK6, TRSK, and iPS-S12 showed up-regulation of pluripotency-related and early embryo-related genes (Fig. 4a). To evaluate the relationship between Cm PSCs and the cells of Cm monkey epiblast (cyEPI) lineage, we examined the expression patterns of cyEPI ontogenic genes, which are able to delineate the stage along with the epiblast development precisely (Fig. 4c,d). Compared with naïve-like Cm ESCs^17^ or our primed-state PSCs, some of our naïve-like PSCs tended to resemble the expression profile of pre-implantation epiblast (Pre-EPI) cells. The correlation coefficient for naïve-like CMK6 cells was high at 0.404. However, all of the naïve-like converted PSCs showed the closest correlation with post-implantation embryos, reflecting an incomplete conversion to the naïve state. These results indicated that our culture system partly succeeded in conversion of a primed state to a naïve state; however, it could not derive true naïve PSCs, which are known to show the expression profile closest to pre-implantation embryos.

### Evaluation of chimera contribution by transferring naïve-like Cm ESCs into host embryos

**Figure 5:**
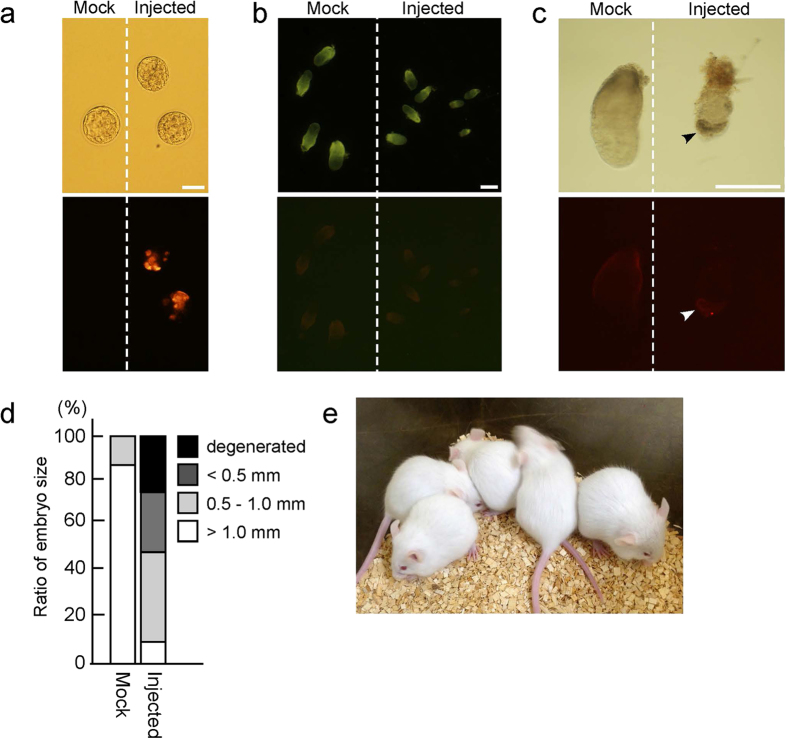
Production of cynomolgus monkey and mouse interspecific chimeras. (**a**) Interspecific blastocysts generated 2 days after injection of naïve-like Cm ESCs (CMK6) into mouse 8-cell embryos. DsRed signals were observed in injected embryos. Mock: mock injected embryo. Scale bar = 100 μm. (**b**) Evaluation of 6.5-dpc embryos. Almost all injected embryos retrieved were retarded in development without any DsRed signals. Scale bar = 500 μm. (**c**) An injected embryo retardedly developed at 6.5 dpc showed Cm ESCs contribution as dark brown colour (black arrowhead) and weak DsRed signal (white arrowhead) in the epiblast. Scale bar = 500 μm. (**d**) Comparison of embryo size of mock-injected and naïve-like ESC-injected embryos recovered at 6.5 dpc. (**e**) Detection of chimeric contribution by brown hair colour. No chimeric contribution was observed in any of the pups examined.

Recently, naïve-like ESCs have been generated from Cm and could produce allogeneic chimeric embryos, albeit showing transcriptomal insufficiency compared with true naïve conversion^17^. To examine whether our naïve-like ESCs would contribute to interspecific chimeras as shown by Fang *et al*.^18^, they were injected into mouse pre-implantation embryos. When more than 10 naïve-like Cm ESCs were injected into mouse 8-cell embryos, almost all the cultured embryos degenerated within 2 days after injection; however, when <5 cells were injected into 8-cell embryos, 27/45 (60%) embryos developed to blastocysts (Fig. 5a and Table 1). The chimeric contribution of Cm ESCs to these mouse blastocysts was clearly shown by the presence of dark-brown cells with DsRed fluorescence (see Supplementary Fig. S8). However, failed or ambiguous integration to the embryos was observed when primed-state CMK6 was injected into 8-cell embryos, as done previously for primed-state rabbit PSCs^12^. When naïve-like Cm ESCs were injected into mouse 8-cell embryos (5 cells/embryo) and then transplanted into the oviducts of pseudopregnant host mothers, almost all the recovered embryos (25/28) had degenerated by the middle of pregnancy (12.5 dpc; see Supplementary Fig. S8 and Table 1). To ascertain when degeneration of interspecific Cm/mouse embryos occurred, 6.5-dpc interspecific chimeric embryos were recovered from the uterus 6 days after transplantation. About a half (37/78; 47%) of the embryos had not implanted or had disappeared. The embryos retrieved were delayed developmentally at around the 5.5-dpc mouse embryo stage with hardly any ESC contribution (Fig. 5b–d). Actually, one 6.5-dpc interspecific chimeric embryo showing dark-brown cells with weak DsRed signals in the epiblast was also retarded (Fig. 5c).

When naïve-like Cm ESCs were injected into early mouse blastocysts (10 cells/embryo) and transplanted into mouse uteri, 37/77 (48%) of the transplanted embryos developed normally without any chimeric contribution even when they were recovered at an early post-implantation stage (6.5 dpc.). No evidence for chimeric contribution (hair colour and DsRed signal) could be detected in any of the pups examined (Fig. 5e). These results suggested that when 8-cell mouse embryos were used as hosts, naïve-like Cm ESCs could contribute temporarily to interspecific chimeras. However, the contribution of Cm ESCs caused developmental impairment to mouse embryos at an early post-implantation stage, possibly because of species differences. On the other hand, when mouse blastocysts were used as recipients, almost all the host embryos could develop normally to term. Recently, Mashaki *et al*. revealed that *E-CADHERIN (CDH1*) and *BCL2* play important roles in chimeric contribution by PSCs^25^. In this context, naïve-like CMK6 enhanced the expression of *E-CADHERIN*, but failed to express *BCL2* (Fig. 1c). These results suggest that naïve-like CMK6 can contribute to pre-implantation embryos; however, naïve-like CMK6 underwent apoptosis as donor PSCs in recipient embryos. This could be why naïve-like ESCs could not survive in chimeric embryos. These results possibly reflect the limited temporal ability of naïve-like ESCs to contribute to chimeric embryos.

### Naïve-like converted PSCs exhibited enhanced ability for *in vitro* neural differentiation

**Figure 6:**
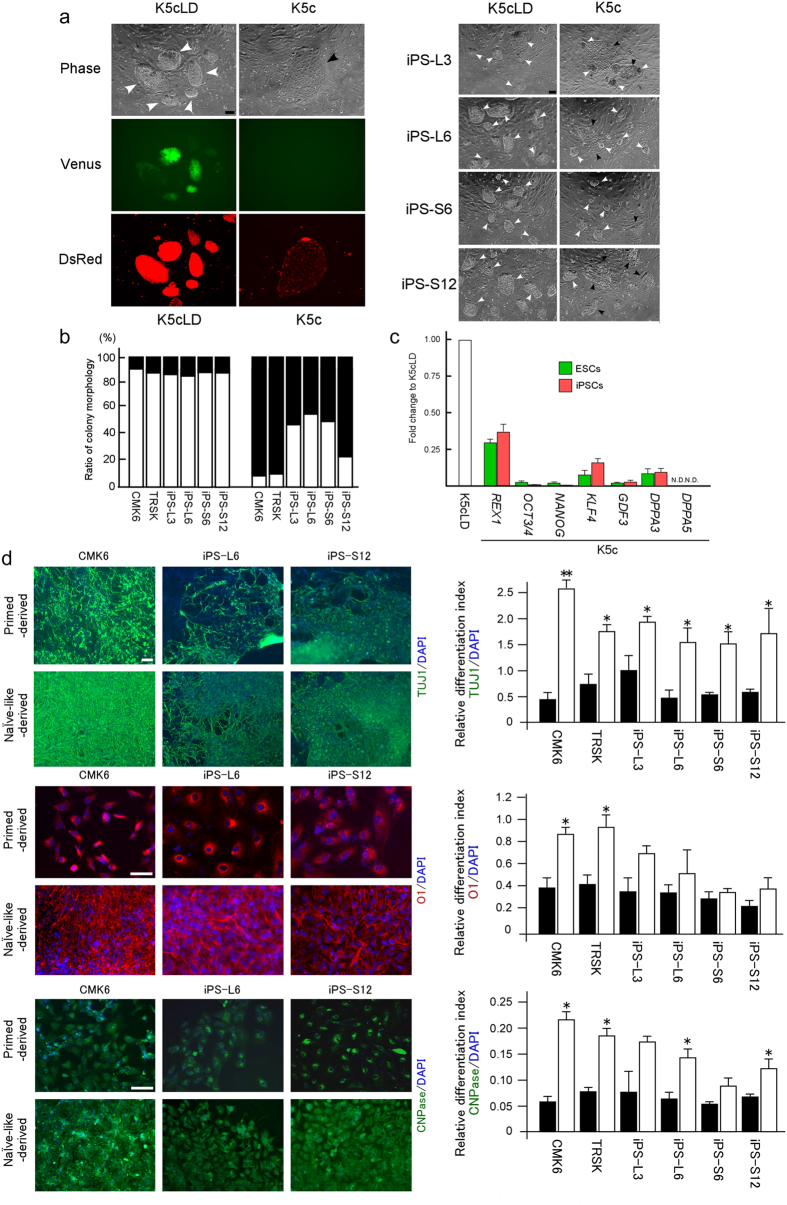
Difference in *in vitro* differentiation potential between naïve-like ESCs and iPSCs. (**a**; left panels) Phase contrast images (Phase), tet-Venus (Venus), and CAG-DsRed (DsRed) signals of a naïve-like converted Cm ESC line, TRSK, before (K5cLD medium) and after (K5c medium) induction of *in vitro* differentiation. Almost all colonies showed a cobble-stone-like cell morphology (black arrowhead) within 10 days after the withdrawal of Dox and LIF. (**a**; right panels) Phase contrast images of the naïve-like converted Cm iPSC lines before (K5cLD medium) and after (K5c medium) the induction of *in vitro* differentiation. In contrast to ESCs, dome-shaped colonies (white arrowheads) remained after 10 days’ withdrawal of Dox and LIF. (**b**) Ratio of colony morphology of naïve-like ESCs and iPSCs before (K5cLD medium) and after (K5c medium) withdrawal of Dox and LIF. Open bars, dome-shaped colonies; closed bars, cobble-stone-like colonies. (**c**) Relative mRNA expression of pluripotency-related genes evaluated by RT–qPCR summarized for naïve-like Cm ESC lines (CMK6, TRSK, and CMK9) and naïve-like iPSC lines (iPS-L3, iPS-L6, iPS-S6, and iPS-S12) before and after withdrawal of Dox and LIF. (**d**) Immunocytochemical evaluation of the *in vitro* ability to differentiate into neurons (TUJ1) and oligodendrocytes (O1 and CNPase) before and after naïve-like conversion of Cm ESCs and iPSCs. Relative neural differentiation indices are shown for neurons immunostained for TUJ1 (top panels and top-right graph), oligodendrocytes marked by O1 (middle panels and middle-right graph) and CNPase (bottom panels and bottom-right graph) derived from Cm ESCs (CMK6, TRSK) and iPSCs (iPS-L3, iPS-L6, iPS-S6, iPS-S12). Closed bars, primed PSC-derived, open bars; naïve-like PSC-derived. Error bars indicate the S.D. *P < 0.05; **P < 0.01. Scale bar = 100 μm.

Previously, we have evaluated neural differentiation ability before and after naïve-like conversion of rabbit PSCs^12^,^13^. Rabbit PSCs show enhanced capacity for neural differentiation after naïve-like conversion. To elucidate the stem cell potential of Cm PSCs more precisely, an *in vitro* neural differentiation assay was also applied before and after naïve-like conversion. Naïve-like converted Cm ESCs reacted specifically to LIF and Dox (Fig. 2). To examine the sensitivity of LIF and Dox in naïve-like iPSCs, initial differentiation following the removal of these factors from the naïve-like iPSC culture conditions was evaluated. Within a week (one passage), almost all colonies of naïve-like ESCs formed a cobble-stone-like morphology (Figs 2a and 6a). However, naïve-like iPSC lines retained some dome-shaped colonies even after the removal of LIF and Dox (Fig. 6a,b). Hardly any differences in the mRNA expressions of pluripotency-related genes appeared between ESCs and iPSCs after the withdrawal of Dox and LIF (Fig. 6c and Supplementary Fig. S7). These results indicate that sensitivity to the removal of LIF and Dox reflects differences in the *in vitro* differentiating ability of naïve-like converted PSC lines. To ascertain whether naïve-like iPSCs would differentiate in a manner similar to naïve-like ESCs, we used an *in vitro* neural differentiation system for forming neurons and oligodendrocytes: the definitive assay for innate differentiation ability in PSCs^12^,^13^. We compared the *in vitro* neural differentiating ability of Cm ESCs (CMK6 and TRSK) and iPSCs (iPS-L3, iPS-L6, iPS-S6, and iPS-S12) before and after naïve-like conversion (Fig. 6d). The *in vitro* neural differentiation capacity of all these cell lines increased more than that of the primed-state PSCs. All of them effectively differentiated into oligodendrocytes with ramified branches, which were not observed even when primed-state cells differentiated (Fig. 6d). Although naïve-like ESCs showed a significantly enhanced differentiation capacity, some of the naïve-like iPSC lines exhibited only limited differentiating ability (Fig. 6d and Supplementary Fig. S9). Transcriptome analysis revealed that candidate genes for neural progenitors (*ZNF521, OTX2, ZIC2, SOX3, PAX6*, and *NES*) did not tend to shift their expression profiles after conversion (Fig. 4a). These results suggest that these naïve-like converted PSCs tended to accelerate their *in vitro* capacity for neural differentiation, despite only partial achievement of naïve conversion.

## Discussion

Our study had four major findings. First, Cm PSCs could be converted into a naïve-like state with several characteristics of naïve pluripotency including colony morphology, increased expression of pluripotency related genes, LIF dependency, and mitochondrial alterations. Second, transcriptome analysis based on pre- and post-implantation Cm embryos revealed that naïve-like converted PSCs had a transcriptomal status most resembling that of post-implantation embryos but tended to shift to the status of pre-implantation embryos. Third, these naïve-like converted Cm ESCs failed to contribute to mouse embryos as interspecific chimeras and showed developmental retardation at just after the implantation stage. Finally, these naïve-like converted PSCs tended to accelerate their *in vitro* capacity for neural differentiation. To derive true naïve PSCs with higher differentiation potential and similar expression profiles to pre-implantation embryos could enable the ability to generate whole organs mainly constituted from donor PSCs in the host animal’s body^26^,^27^. Further examinations that focus on their transcriptomal status without miscellaneous naïve-like characteristics will be a key goal for deriving true naïve PSCs in non-human primates.

Several reports have demonstrated the conversion of primed human PSCs to a naïve (-like) state using different strategies^15^,^16^,^28^,^29^. The naïve status of PSCs in these reports was determined by several morphological and molecular similarities to mouse ESCs^7^,^14^. However, because of the ethical impediments, one of the most stringent criterions of true naïve pluripotency—the ability to contribute to chimeric embryos or pups—rarely been examined using naïve (-like) human PSCs. To determine their naïve pluripotency more precisely, their transcriptome status after naïve conversion should be assessed to evaluate whether they resemble those of human pre-implantation embryos. Actually, some human PSCs showed a transcriptome status resembling human pre-implantation embryos^30^,^31^. Here, naïve-like Cm ESCs barely remained in mouse embryos as interspecific chimeras at just after implantation, and they exhibited developmental blocks. Some reports have raised the possibility that the ability to contribute to chimera formation is not the best criterion of true naïve pluripotency. Actually, some human and non-human primate PSCs have demonstrated naïve-like conversion and also achieved the ability to contribute to interspecific chimeras^17^,^28^. However, transcriptome evaluations with these pre- and post-implantation embryos showed incomplete naïve-like conversion^7^,^14^. On the other hand, human naïve ESCs most resemble mouse ESCs and primate pre-implantation embryos but are only incorporated into mouse embryos as interspecific chimeras very inefficiently^28^. Interspecific chimera formation using PSCs has been achieved in the mouse and rat^20^,^26^,^32^. On the other hand, if truly naïve PSCs from other mammalian species could be generated successfully, it is not certain whether they could contribute to interspecific chimeras efficiently or not. Here, when Cm naïve-like ESCs were injected into mouse 8-cell embryos, almost all the chimeric embryos degenerated or were retarded around the implantation stage. These results imply that naïve-like Cm ESCs contributed temporarily to the mouse embryos but had negative effects at around 5.5–6.5 dpc. On the other hand, when mouse blastocysts were used as hosts, naïve-like Cm ESCs were barely incorporated at all, and the embryos developed normally without any developmental impairments. These results suggest that the generation of chimeras using other mammalian PSCs needs to be examined, for not only the status of PSCs but also for host embryo compatibilities in terms of the species involved and their developmental stages.

Enhanced ability for *in vitro* neural differentiation was demonstrated after naïve-like conversion of Cm PSCs even if they did not completely reach true naïve pluripotency. We also found that these naïve-like ESCs showed superior capacity for oligodendrocyte differentiation than iPSCs. We demonstrated previously that naïve-like conversion enhances the difference in innate *in vitro* differentiation capacity between rabbit ES cells and iPS cells^13^. Almost no *in vitro* differentiation targets were achieved, other than the formation of germ cells, which can be generated more effectively in naïve PSCs than in primed PSCs^6^,^12^,^13^. Although evaluations of targeted *in vitro* differentiation potential with several target lineages before and after naïve conversion of human PSCs need to be demonstrated, the *in vitro* targeted induction of differentiation by confirming the potential for forming chimeras needs to be examined using experimental animals. Non-human primate PSCs, which transcriptionally most resemble pre-implantation epiblasts, are needed for assessing whether they can contribute effectively to chimeras, and can differentiate efficiently to desired target cell types *in vitro*.

In conclusion, we have attempted to convert primed-state Cm PSCs to a naïve state. Despite our confirmation of several naïve characteristics, transcriptome evaluation suggested that they should be classified not as “true naïve state” but as having an incomplete “naïve-like” state. We have also elucidated that these naïve-like PSCs showed enhanced ability for *in vitro* neural differentiation. The achievement of true naïve conversion using non-human primate PSCs will lead to practical applications in human regenerative medicine.

## Materials and Methods

### Animals

All animals (Cm and ICR mice) were maintained and used for experiments in accordance with the guidelines for animal experimentation of the University of Miyazaki, RIKEN Bioresource Center, and Tsukuba Primate Research Center after obtaining approvals by the responsible committees of each institution.

### Naïve-like conversion of Cm ESCs

The Cm ESC lines CMK6 (male), CMK9 (female), and TRSK (male) were used as reported^33^,^34^. To maintain their primed-state pluripotency, Cm ESCs were cultured in bFGF/KSR medium consisting of 78% Dulbecco’s modified Eagle’s medium/Ham’s F-12 (DMEM/F12) supplemented with 15% KSR, 2 mM GlutaMax (Invitrogen Life Sciences, Carlsbad, CA, USA), 1% non-essential amino acids, 0.1 mM mercaptoethanol, and 4 ng/ml human recombinant bFGF (Wako, Osaka, Japan). To convert Cm ESCs into a naïve-like state, PiggyBac (PB) vectors carrying Dox-inducible *hKLF2* or *hNANOG* coupled to Venus were cotransfected with a PB-EOS-*GFP/puroR* reporter, which has an rtTA expression construct^15^,^20^, and pCAG-PBase using an NEPA21 electroporator (Nepa Gene Co., Ltd., Chiba, Japan). To confirm the chimeric contribution of ESCs into mouse embryos as interspecific chimeras, cell lines transfected with PB-CAG-*Su9DsRediN* were established concurrently. One day after transfection, G418 (200 μg/ml) and puromycin (250 ng/ml) were added to the selection in the presence of 1 μM Dox. Two weeks after selection, transfectants were dissociated with trypsin digestion and replated and cultured in naïve-like conversion medium (K5cLD) consisting of 38% DMEM/F12 and Neurobasal Medium supplemented with 20% KSR, 1% N2 supplement, 2% B27 supplement, 2 mM GlutaMax, 1% non-essential amino acids (Invitrogen Life Sciences), 0.1 mM β-mercaptoethanol, 1% penicillin-streptomycin, and a five-chemical mix (5c): 5 μM Gö6983, 10 μM forskolin (Sigma-Aldrich, St. Louis, MO, USA), 5 μM kenpaullone, 3 μM CHIR99021 (Stemgent Inc., Cambridge, MA, USA), 0.5 μM SB590885 (Wako). Additionally, cell lines were evaluated in the presence or absence of 0.1% recombinant human LIF (L in media abbreviations; Wako) and 1 μM Dox (D in media abbreviations; TaKaRa, Bio Inc., Shiga, Japan). K5cLD was changed daily, and cells were passaged using trypsin/EDTA by single cell dissociation and replated on mitomycin C-treated mouse embryonic fibroblast (MEF) feeder cells. To determine the appropriative culture conditions for Cm naïve conversion, a culture medium used for rabbit cell naïve-like conversion^12^ was also assessed using K5cLD in the absence of Gö6983 and SB590885 (this medium was termed K3cLD).

To assess the culture media reported by Takashima *et al*.^15^, PB-tet-*KLF2*, PB-tet-*NANOG*, PB-EOS-*GFP/puroR*, and PB-CAG-*Su9DsRediN* were transfected into the Cm ESC line, CMK6, and cultured for 2 weeks in conventional primed culture medium in the presence of 1 μM Dox. According to Takashima *et al*., to derive Cm naïve PSCs, cultures were switched to *t* (titrated) 2iLD medium consisting of 48% DMEM/F12, 48% Neurobasal medium, 1% N2 supplement, 2% B27 supplement, 2 mM GlutaMax (Invitrogen Life Sciences), 0.1 mM β-mercaptoethanol, 1% penicillin-streptomycin, small molecules—1 μM CHIR99021, and 1 μM PD0325901—(Stemgent), and 0.1% recombinant human LIF in the presence of Dox. Human-embryo-derived H9 cells that overexpressed *NANOG* and *KLF2* genes were cultured in *t*2iLD medium for evaluating Cm naïve conversion.

To assess LIF-dependent maintenance of the naïve-like pluripotency of Cm ESCs, CMK6 and TRSK cell lines were cultured on DR4 feeder cells (Applied StemCell, Inc., Milpitas, CA, USA) expressing the puromycin resistance gene. The culture medium used was K5cL (in the presence of LIF) or K5c (in the absence of LIF). Cells were treated with 0.5 μg/ml of puromycin for 10 days.

### iPS cell generation and culture

Cultured Cm somatic cells were trypsinized to disperse into single cells and electroporated with 10 μg each of pCXLE-hOCT3/4-shp53-F, pCXLE-hUL, or pCXLE-hSK^35^ (Addgene, Cambridge, Massachusetts, USA) using a NEPA21 electroporator. Transfected cells were cultured at 37 °C under 6% CO_2_ in air using DMEM supplemented with 10% foetal bovine serum, penicillin, and streptomycin. Eight days after transfection, cells were replated into 100-mm culture dishes and cultured using bFGF/KSR medium on MEF feeder cells at 37 °C under 6% CO_2_ in air. Twenty-six days after electroporation, iPS-like cell colonies were isolated mechanically and replated onto MEF feeder cells. Passaging of Cm iPSCs was performed by incubating the cells with phosphate buffered saline supplemented with 0.25% trypsin, 1 mg/ml collagenase type IV, 20% KSR, and 1 mM CaCl_2_. Fresh medium was changed daily, and cells were passaged every 3–4 days. To convert iPSCs into a naïve-like state, plasmids vectors for naïve-like conversion of Cm ESCs other than PB-CAG-*Su9DsRediN* were transfected and cultured as above. The Cm PSC lines used could be divided roughly into three categories of cell lines: embryonic stem (ES: CMK6 (male), CMK9 (female), and TRSK (male)); liver-derived iPS (iPS-L3, iPS-L6); and stomach-derived iPS (iPS-S6, and iPS-S12). After establishment, to confirm persistence of the remaining exogenous plasmid vectors, genomic DNA samples of all iPSC lines were subjected to genomic PCR using the specific primers listed in Supplementary Table S4.

### Total RNA preparation and mRNA expression analysis

To avoid contamination from MEF feeder cells, Cm PSC lines (CMK6, TRSK, CMK9, iPS-L3, iPS-L6, iPS-S6, and iPS-S12) were cultured for more than two passages on MEF-free culture plates coated with laminin-511 (Wako). Total RNA was isolated with RNeasy Plus mini kits (QIAGEN, Hilden, Germany) from cells cultured under the appropriate conditions. After QIAshredder (QIAGEN) treatment to prevent contamination with genomic DNA, the first-strand cDNA was synthesized using RNA PCR kits (TaKaRa Bio Inc.) for reverse transcriptase polymerase chain reaction (RT–PCR). The synthesized cDNA was amplified by PCR using the specific primers listed in Supplementary Table S4, with a cycling program of 94 °C for 3 min and 35 cycles of 94 °C for 30 s, 60 °C for 30 s, and 72 °C for 30 s. For RT–qPCR, a LightCycler 96 (Roche Applied Sciences, Mannheim, Germany) was used to determine mRNA expression levels using the Fast Start Essential DNA Green Master Mix (Roche Applied Sciences), with a program of 94 °C for 10 min, 40 cycles of 94 °C for 10 s, 60 °C for 10 s, and 72 °C for 10 s.

### Transcriptome analysis by SC3-Seq

Total RNAs of Cm ESCs (CMK6, TRSK, CMK9) and iPSCs (iPS-L3, L6, S6, S12), cultured in bFGF/KSR (primed), K5cLD (naïve-like), or K5c (differentiated) media, were prepared as above. Total RNAs of Cm somatic cells from the liver and stomach were also prepared. cDNA synthesis and the construction of a SC3-seq library were carried out as reported^7^,^36^. Briefly, cDNA was synthesized from 1 ng of total RNA s and evaluated for quality using an ERCC RNA Spike-In Mix (Ambion #4456740; Thermo Fisher Scientific). SC3-seq libraries of quality-checked cDNAs were constructed and sequenced using a Nextseq500 system (Illumina, San Diego, CA, USA; 75 bp, single read; FC-404-2005). Expression levels were converted using cufflinks v. 2.2.0^37^ with the “-compatible-hits-norm-library-type fr-unstranded-max-mle-iterations 50000” options. We defined expressed genes as those whose log_2_ (RPM + 1) values were >4 or greater than ~10–20 copies per cell, which is a lower limit for reliable and reproducible detection by the single-cell cDNA amplification method^7^,^36^ in at least one sample. UHC was performed using the “hclust” function with Euclidian distances and Ward’s method (ward.D2). PCA was performed using the “prcomp” function without scaling. Gene expression of the PSCs in several culture conditions were compared with those of Cm embryos in the undifferentiated inner cell mass (ICM), pre-implantation epiblasts (Pre-EPI), post-implantation early epiblasts (PostE-EPI), post-implantation late epiblasts (PostL-EPI), and gastrulating cells 1, 2a, and 2b (Gast1, 2a, and 2b)^7^.

### Evaluation of chimeric contribution to mouse embryos

To evaluate whether the naïve-like Cm ESCs could contribute to mouse embryos and pups as interspecific chimeras, naïve-like CMK6 or TRSK cells were trypsinized to dissociate into single cells. Recipient embryos were obtained by *in vitro* fertilization using superovulated ICR strain mouse oocytes and ICR sperm in human tubal fluid medium (ARK Resource, Kumamoto, Japan) and then cultured in potassium simplex optimized medium (ARK Resource) to develop into 8-cell stage embryos or blastocysts. Naïve-like CMK6 or TRSK cells (*n *=* *5–10) were injected into the perivitelline spaces of 8-cell embryos or blastocoels of the blastocysts using a Piezo-driven micromanipulator (Prime Tech Ltd., Ibaraki, Japan). Interspecific 8-cell embryos or blastocysts were transplanted into the oviducts or uteri of pseudopregnant ICR mice, respectively. Early post-implantation (5.5- and 6.5-dpc) embryos were recovered and observed using a fluorescence microscope, MVX10 (Olympus, Tokyo, Japan). Chimerism of any embryos or neonates was determined by the presence of DsRed signals and brown hair colour.

### *In vitro* neural differentiation

As reported^12^,^13^ to induce neural differentiation, Cm PSC lines were digested with trypsin, suspended in embryoid body (EB) medium containing 78% DMEM/F-12 supplemented with 20% KSR, 1% non-essential amino acids, 50 units/ml penicillin, 50 μg/ml streptomycin, 0.1 mM β-mercaptoethanol, 1% N2 supplement, 4 μM all-*trans*-retinoic acid (Sigma-Aldrich), and 10 μM SB431542 (Tocris Bioscience, Bristol, UK). To achieve single EBs of a uniform size, 1,000 PSCs in a volume of 100 μl were dispensed into each well of low-cell-adhesion 96-well round-bottomed plates (Thermo Fisher Scientific, Waltham, MA, USA), tapped gently, and cultured at 37 °C under 6% CO_2_ in air. To obtain differentiated oligodendrocytes, six EBs were transferred to matrigel-coated (BD Biosciences, Franklin Lakes, NJ, USA) 4-well multi-dishes (Nunc) and allowed to attach to the bottoms of the wells. The medium was then changed from EB medium to neural differentiation medium (the same formulation as EB medium, with the addition of 10% KSR). After 10 days, the medium was changed from neural differentiation medium to the culture medium without retinoic acid or SB431542 but with 100 ng/ml Noggin (Wako). The cells were cultured for a further 20 days, with fresh medium changed every day. After *in vitro* neural differentiation, cells were immunochemically stained using the neural antibodies anti-TUJ1, anti O1, and anti-CNPase.

### Statistical analysis

Mean values were compared using one-way analysis of variance (ANOVA). Where appropriate, the significance of differences in the means was determined with Fisher’s exact probability test. P < 0.05 was considered to be significant. All experiments were analysed at least in triplicate.

## Additional Information

**Accession codes**: SC3-seq. data have been deposited in the Gene Expression Omnibus database (GEO; http://www.ncbi.nlm.nih.gov/geo/) and given the series accession number GSE87576.

**How to cite this article**: Honda, A. *et al*. Discrimination of Stem Cell Status after Subjecting Cynomolgus Monkey Pluripotent Stem Cells to Naïve Conversion. *Sci. Rep.* **7**, 45285; doi: 10.1038/srep45285 (2017).

**Publisher's note:** Springer Nature remains neutral with regard to jurisdictional claims in published maps and institutional affiliations.

## Supplementary Material

Supplementary Information

## Figures and Tables

**Table 1:** Generation of naïve-like Cm ESCs–mouse interspecific chimeras.

Cell line	Host embryos (injected cells/embryo) 8-cells blastocysts		*In vitro* blastocysts (DsRed+) (degenerated or retarded)	Transplanted (pregnancy/foster mother)		Implanted (DsRed+)/Transplanted (degenerated or retarded)		
	(5)	(10)		oviduct	uterus	6.5 dpc	12.5 dpc	pups
CMK6	123		15 (9) (4)	95 (4/5)		23(0)/40 (21)	15(0)/35 (13)	n.d.
		135			118 (5/6)	30(0)/40 (8)	30(0)/38 (4)	13(0)/20 (4)
TRSK	95		12 (6) (6)	78 (3/4)		18(1)/38 (16)	13(0)/20 (12)	n.d.
		97			97 (4/5)	19(0)/37 (4)	10(0)20 (2)	12(0)/20 (4)
total	218	232	27 (15) (10)	173 (7/9)	215 (9/11)	90(1)/155 (49)	68(0)/113 (31)	25(0)/40 (8)

n.d: not determined.
