# Supplementary material for: Discrimination of Stem Cell Status after Subjecting Cynomolgus Monkey Pluripotent Stem Cells to Naïve Conversion
Source: Sci Rep. 2017 Mar 28;7:45285. doi: 10.1038/srep45285 (PMC5368663; doi:10.1038/srep45285)
Supplement: Supplementary Information [file srep45285-s1.doc]

# Supplementary Information

Discrimination of Stem Cell Status after Subjecting Cynomolgus Monkey Pluripotent Stem Cells to Naïve Conversion

Arata Honda^1,2*^, Yoshihiro Kawano^1^, Haruna Izu^1^, Narantsog Choijookhuu^3^ , Kimiko Honsho^1^, Tomonori Nakamura^4,5^, Yukihiro Yabuta^4,5^, Takuya Yamamoto^6,7,8^, Yasuhiro Takashima^6^, Michiko Hirose^2^, Tadashi Sankai^9^, Yoshitaka Hishikawa^3^, Atsuo Ogura^2^, Mitinori Saitou^4,5,6,7^

^1^ Organization for Promotion of Tenure Track, University of Miyazaki, 5200, Kibara, Kiyotake, Miyazaki 889-1692, Japan.

^2^ RIKEN BioResource Center, Tsukuba, Ibaraki 305-0074, Japan.

^3^ Department of Anatomy, Histochemistry and Cell Biology, Faculty of Medicine, University of Miyazaki, Miyazaki 889-1692, Japan.

^4^ Department of Anatomy and Cell Biology, Graduate School of Medicine, Kyoto University, Yoshida-Konoe-cho, Sakyo-ku, Kyoto 606-8501, Japan.

^5^ JST, ERATO, Yoshida-Konoe-cho, Sakyo-ku, Kyoto 606-8501, Japan.

^6^ Center for iPS Cell Research and Application, Kyoto University, 53 Kawahara-cho, Shogoin, Sakyo-ku, Kyoto 606-8507, Japan.

^7^ Institute for Integrated Cell-Material Sciences, Kyoto University, Yoshida-Ushinomiya-cho, Sakyo-ku, Kyoto 606-8501, Japan.

^8^ AMED-CREST, AMED, 1-7-1 Otemachi, Chiyoda-ku, Tokyo, 100-0004, Japan.

^9^ Tsukuba Primate Research Center, National Institute of Biomedical Innovation, Health and Nutrition, Tsukuba, Ibaraki, 305-0843, Japan.

^*^To whom correspondence should be addressed. Arata Honda, Organization for Promotion of Tenure Track, University of Miyazaki, 5200, Kibara, Kiyotake, Miyazaki 889-1692, Japan. Telephone: 81-985-85-9866. E-mail:a-honda@med.miyazaki-u.ac.jp

**Figure S1. Proliferation and appearance of cynomolgus monkey (Cm) ESC line.** (**a**) A Cm ESC line, CMK6, which was transfected with *hNANOG* and *hKLF2*, was cultured using primed-state condition for 2-weeks (bFGF/KSR medium, black line). After replacement with *t*2iLD medium (blue), Cm ESCs could not be maintained in a manner similar to human naïve hESCs (purple). However, the K5cLD (green) or K3cLD (red) lines could maintain their proliferating ability together with naïve-like characteristics. (b) Colony morphology of Cm ESCs and hESCs cultured in several different media. Arrowheads indicate differentiated colonies. Scale bar = 100 μm.

**Figure S2. Appearance of differentiating colonies in the absence of Dox**. After withdrawal of Dox, putative differentiating colonies (black arrowheads) appeared and increased with culture. Scale bar = 100 μm.

**Figure S3. Immunohistochemical confirmation of teratomas derived from naïve-like converted ESCs**. Sections of teratomas derived from a naïve-like converted ESC line (CMK6). Neurons (top panels) and muscle cells (bottom panels) were confirmed by immunostaining using an anti-neural specific enolase (NSE) antibody and anti α-smooth muscle actin antibody (aSMA), respectively. HE, haematoxylin and eosin staining. Scale bar = 100 μm.

**Figure S4. Reversible generation of prime-type colonies from naïve-like colonies.** Naïve-like CMK6 cells were reversibly converted to a primed state by changing from K5cLD medium to primed culture medium (bFGF/KSR). Scale bar = 100 μm.

**Figure S5. Establishment of iPSCs from Cm somatic cells.** (a) Phase contrast images of somatic cells (liver and stomach; top panels), generated iPSCs (middle panels; the left shows iPS-L6 cells, and the right shows iPS-S12 cells), and their alkaline phosphatase activity (AP, bottom panels). (b) Semi-quantified RT–PCR analysis of the expression of selected pluripotency-related genes (Endo-) and transgenes (Exo-) in Cm iPSC lines, and the original Cm liver and stomach cells. All pluripotency-related genes were expressed, whereas iPS-L3 and iPS-S6 cell lines slightly expressed exogenous *OCT3/4* genes. (c) Detection of exogenous plasmids in iPS lines by genomic PCR. A plasmid vector, pCXLE-hOCT3/4-shp53-F, was detected in the iPS-L3, iPS-L6, and iPS-S12 cells. (d) Teratoma formation with all three germ layers is shown by haematoxylin and eosin staining (neurons and muscle), toluidine blue staining (cartilage), alcian blue staining (goblet cells), and immunostaining (anti NSE and anti-αSMA antibodies). These images represent teratoma sections derived from iPS-S12 cells. Scale bar = 100 μm.

**Figure S6. Immunohistochemical confirmation of teratomas derived from naïve-like converted iPSCs (iPS-S12).** Neurons (left panels) and muscle cells (right panels) were confirmed by immunostaining using anti-NSE and anti-αSMA antibodies, respectively. HE, haematoxylin and eosin staining. Scale bar = 100 μm.

**Figure S7. Expression of naïve pluripotency-related genes of the Cm ESC and Cm iPSC lines before and after the withdrawal of Dox and LIF.** Expression levels of pluripotency-related genes in each cell line after the withdrawal of LIF and Dox (K5c) are shown as relative to naïve culture conditions (K5cLD medium).

**Figure S8. Contribution of Cm ESCs to mouse blastocysts.** (**a**) Naïve-like Cm ESCs observed in chimeric blastocysts are clearly distinguished as dark-brown cells (black arrowheads) and DsRed signals (white arrowheads) (right panels). However, weak signals were detected in the chimeric embryos that had been injected with primed-state Cm ESCs (left panels). Scale bar = 100 μm. (**b**) When naïve-like Cm ESCs were injected into mouse 8-cell embryos, almost all the recovered embryos (25/28) had degenerated at the middle of pregnancy (12.5 dpc).

**Figure S9. Relative oligodendrocyte differentiation index of Cm ESC lines (CMK6 and TRSK) and iPSC lines (iPS-L3, iPS-L6, iPS-S6, and iPS-S12).** Closed bars, primed PSC-derived lines, open bars; naïve-like PSC-derived lines. Error bars indicate the S.D. *P < 0.05.

| **Status** | **Characteristics** | **Importance** |
| --- | --- | --- |
| Pluripotent | 1. Self-renewal activity | +++ |
|  | 2. Expression pluripotency related genes | ++ |
|  | 3. Teratoma (with three germ layers) forming ability | +++ |
| Naïve-like | 4. Domed-shape colony morphology | + |
|  | 5. LIF dependency | + |
|  | 6. Expression naïve pluripotency related genes | ++ |
|  | 7. Naïve pluripotent transcriptomal circuitry | ++ |
|  | 8. Ability to use mitochondrial respiration | ++ |
| Naïve | 9. Transcriptomal status resembling pre-implantation epiblasts | +++ |
|  | 10. Efficient ability to contribute to chimeric embryos with subsequent germline transmission | ++ |

**Supplementary Table S1. Characteristics of naïve-like and naïve pluripotency.**

A naïve pluripotent state fulfils characteristics 1–9, and ideally 10. In contrast, a naïve-like pluripotent state exhibits characteristics 1–8 but not 9 or 10. The relative importance of characteristics is indicated as: +, not necessary but ideally required; ++, required; and +++, necessary.

| Cm PSC lines | Normal/analysis (%) |
| --- | --- |
| CMK6 (P) | 32/40 (80) |
| CMK6 (N) | 34/39 (87) |
| TRSK (P) | 21/25 (84) |
| TRSK (N) | 22/24 (92) |
| iPS-L3 (P) | 20/25 (80) |
| iPS-L3 (N) | 22/28 (79) |
| iPS-L6 (P) | 32/35 (91) |
| iPS-L6 (N) | 37/40 (93) |
| iPS-S6 (P) | 19/23 (83) |
| iPS-S6 (N) | 20/24 (83) |
| iPS-S12 (P) | 37/40 (93) |
| iPS-S12 (N) | 36/40 (90) |

**Supplementary Table S2. Karyotype analysis.**

P, Primed; N, Naïve-like

|  | Cell lines | ECC | AP | IA | RT | Karyo | Tera | LWA | Mito | IND | RNA-seq | ICP |
| --- | --- | --- | --- | --- | --- | --- | --- | --- | --- | --- | --- | --- |
| ESC lines | CMK6 (P) | + | + | n.d. | + | + | n.d. | + | + | + | + | + |
|  | CMK6 (N) | + | + | + | + | + | + | + | + | + | + | + |
|  | TRSK (P) | + | + | n.d. | + | + | n.d. | + | + | + | + | n.d. |
|  | TRSK (N) | + | + | + | + | + | + | + | + | + | + | + |
|  | CMK9 (P) | n.d. | n.d. | n.d. | + | n.d. | n.d. | n.d. | n.d. | n.d. | + | n.d. |
|  | CMK9 (N) | n.d. | n.d. | n.d. | + | n.d. | + | n.d. | n.d. | n.d. | + | n.d. |
| iPSC lines | iPS-L3 (P) | n.d. | + | + | + | + | + | n.d. | n.d. | + | + | n.d. |
|  | iPS-L3 (N) | n.d. | + | + | + | + | + | n.d. | n.d. | + | + | n.d. |
|  | iPS-L6 (P) | n.d. | + | + | + | + | + | n.d. | n.d. | + | + | n.d. |
|  | iPS-L6 (N) | n.d. | + | + | + | + | + | n.d. | n.d. | + | + | n.d. |
|  | iPS-S6 (P) | n.d. | + | + | + | + | + | n.d. | n.d. | + | + | n.d. |
|  | iPS-S6 (N) | n.d. | + | + | + | + | + | n.d. | n.d. | + | + | n.d. |
|  | iPS-S12 (P) | n.d. | + | + | + | + | + | n.d. | n.d. | + | + | n.d. |
|  | iPS-S12 (N) | n.d. | + | + | + | + | + | n.d. | n.d. | + | + | n.d. |

**Supplementary Table S3. Characterization of Cm PSC lines.** P, primed; N, naïve-like; ECC, evaluation of culture conditions; AP, alkaline phosphatase activity; IA, immunocytochemical analysis; RT, RT–qPCR; Karyo, karyotyping; Tera, teratoma formation; LWA, LIF withdrawal assay; Mito, evaluation of mitochondrial status; IND, *in vitro* neural differentiation; ICP, interspecific chimera production using mice; +, examined; n.d., not determined.

| Gene | Forward (5′ to 3′) | Reverse (5′ to 3′) |
| --- | --- | --- |
| Primers | | |
| *OCT3/4* | GGGAGGAGCTAGGGAAAGAGAACCTA | CCCCCACCCGTTGTGTTCCCA |
| *GDF3* | AAATGTTTGTGTTGCGGTCA | TGAAGGGAGCGTCTCAGTCT |
| *DNMT3L* | CTGCTGACTCAGAACAGGCA | GTGACGACGCAAAAACCGAA |
| *DPPA5* | TTCCCGAAGACCTGAAAGATCC | CCGGGCCAAACATGGCTTTC |
| *KLF4* | TTGGGGTTTTCGGTTTTGGC | GCGAACGTGGAGAAAGATGG |
| *KLF5* | GACACCTCAGCTTCCTCCAG | TGGGATTTGTAGAGGCCAGT |
| *SOX2* | AAGGTTTTTCCCCCTTTATTTTCCG | GATTCTCGGCAGACTGATTCAAATA |
| *TBX3* | TGCTGATTTTTCGCCTTGCC | TCTGCAGCTGCTGTGTTTTG |
| *TFCP2L1* | TCCCATTTTTCTGGCATGCC | ATGAAACCTGGAGCTTTCGC |
| *CDH1* | ATGACAACAAGCCCGTGTTC | TTTTTGTCAGGCAGCTCAGG |
| *ESRRB* | AATGCCCCTTTTGGATCAGC | TGGAAAAGCTGCAGAAAGGC |
| *BCL2* | TCGGGCAACAGAAAACCATC | ATTTGTGCAGCAAGGGACTG |
| *NANOG* | TGTTCCGGTTTCCATTATGCC | TAGGCTCCAACCATACTCCA |
| *KLF2* | TGAGAAACCCTACCACTGCA | AAGGAGGATCGTGGTCTTTTCC |
| *Cox6b1* | GCTGGCAGAACTACCTGGAC | CATCCTGGGAGAAGGACAGA |
| *Cox7a1* | GAGTGCCCGAGAAACAGAAG | TCAGGTACAACGGGATGTCA |
| *Cox8c* | ATGGCTGTTGGACTTGTGGT | CCACAATGGTTGCATCACTC |
| *Nqo1* | CAGATCCTGGAAGGATGGAA | AAGTGATGGCCCACAGAAAG |
| *CAMP* | GCAGACCAGCATCTCAGACA | CAGAGTTTTCTTTCCGGCATT |
| *REX1* | ACCACTGTATCAAACTATATGGAACT | AAATGGATTCAAATGGACTAACGCT |
| *DPPA3* | AGTTTCTGCGTGTCCAATGG | GCATAGAGTAGCTTTCACAACCT |
| *Exo-hKLF2* | GCGCTCAGACGAGCTCACG | CATGTGCCGTTTCATGTGCAG |
| *Exo-hNANOG* | TTCTGCAGAGAATAGTGTCG | CGTCACACCATTGCTATTCTTC |
| *ACTB* | AGACCTGTACGCCAACACAG | AGGGCCAGACTCGTCATACT |
| Endo-primers | | |
| *NANOG* | CCTATGCCTGTGATTTGTGGG | AGGTTGTTTGCCTTTGGGAC |
| *OCT3/4* | CAGATCAGCCACATCGCCCAG | CAGATCAGCCACATCGCCCAG |
| *KLF4* | TCGTTGAACTCCTCGGTCTC | CTCTCCCACATGAAGCGACT |
| *SOX2* | GGTTACCTCTTCCTCCCACTCC | CCTCCCATTTCCCTCGTTTT |
| *L-MYC* | AGATTGCAAAGGTCGACTGC | ACCGGTGCATTTTCTTCATC |
| *G3PDH* | ACCACAGTCCATGCCATCAC | TCCACCACCCTGTTGCTGTA |
| Exo-primers | | |
| *OCT3/4* | CAGAAGGGCAAGCGATCA | ATTCCTAGAAGGGCAGGCA |
| *KLF4* | CATGAAGCGACTTCCCCCA | GCAAGCCGCACCGGCTC |
| *L-MYC* | AGCGAGGACATCTGGAAGAA | CAGCTTTCTGGAGGAAAACG |
| *pCXLE-*  *hOCT3/4-shp53-F* | CCATTTTGGTACCCCAGGCT | AAAGTCCCGGAAAGGAGCTG |
| *pCXLE-hUL* | ATGGTAATCGTGCGAGAGGG | ATCCTCCCCGCAGTCATAGT |
| *pCXLE-hSK* | TTGTCCCAAATCTGTGCGGA | CCGGGCTGTTTTTCTGGTTG |

**Supplementary Table S4. PCR primers used for RT–PCR.** Endo-primers, specifically recognized Cm cDNAs; Exo-primers, detect Tg-specific sequences.

**Supplementary Materials and Methods**

**Cell lines and culture details.** Somatic cells derived from the liver and stomach of a female Cm foetus (79 days of gestation) were extirpated, incised, washed with Hanks’ balanced salt solution, and bluntly dissected using 1 mg/ml collagenase, 1.4 mg/ml DNase, and 0.2% trypsin. After termination of enzyme treatment by adding foetal bovine serum (FBS), loosened tissues were dissected into single cells and small clumps by gentle pipetting. These cells were plated and cultured in Dulbecco’s modified Eagle’s medium (DMEM) supplemented with 10% FBS, 10 mg/l insulin, 5.5 mg/l transferrin, 6.7 μg/l selenium, 40 ng/ml hepatocyte growth factor (Peprotech, Rocky Hill, NJ, USA), 20 ng/ml epidermal growth factor (Peprotech), penicillin, and streptomycin. The H9 human embryo-derived cell line was used (WiCell Research Institute, Madison, WI, USA)^1^.

**Plasmid preparation.** To prepare pPB-CAG-Su9DsRed-IRES-NeoR, the Su9DsRed2 fragment was excised from pCXN-Su9DsRed2[^2^](#_ENREF_1) using the restriction endonuclease *Eco*RI. The Su9DsRed2 fragment was introduced into the *Eco*RI site of pPBCAG-cHA-IN.

**Immunocytochemical analysis.** Cm PSC lines (CMK6, TRSK, iPS-L3, iPS-L6, iPS-S6, and iPS-S12), which were not transfected with *DsRed* gene, were used for immunocytochemical analysis. Marker expression was analysed by fixing the cells attached to the bottoms of the culture plates with 4% paraformaldehyde for 30 min at room temperature and then washing them three times (5 min each) with Tris-buffered saline containing 1% bovine serum albumin (BSA; wash buffer). To permeabilize the cells, they were treated with 0.1% Triton X-100 in wash buffer for 10 min and then incubated in blocking solution (10% normal donkey serum and 1% BSA in wash buffer) for 30 min. The following primary antibodies were used: anti-OCT3/4, anti-SOX2, anti-KLF4, anti-REX1, and anti-O1 from Santa Cruz Biotechnology (Dallas, TX, USA), anti-NANOG from COSMOBIO (Tokyo, Japan), anti-KLF17 from Atras Antibodies, AB, Bromma, Sweden), anti-TUJ1 from R and D systems, Inc. (Minneapolis, MN, USA) and anti-CNPase from Sigma-Aldrich (St. Louis, MO, USA). All antibodies were diluted in blocking solution and incubated with the samples overnight at 4 °C. The next day, the cells were washed three times with wash buffer and incubated with secondary antibodies at room temperature for 1 h. The cells were washed three times with wash buffer and covered with 50% glycerol containing DAPI. The fluorescent signals were detected and quantified using a BZ-9000 Series All-in-One Fluorescence Microscope and BZII image analysis system (Keyence, Osaka, Japan).

**Karyotyping.** Cm PSC lines (CMK6, TRSK, iPS-L3, iPS-L6, iPS-S6, and iPS-S12) before and after naïve-like conversion were incubated with colcemid (final concentration 100 ng/ml) for 4 h at 37 °C in 6% CO_2_ in air. Cells were trypsinized and pelleted at 120 *g* for 3 min, resuspended in 6 ml of 75 mM KCl, and incubated at 37 °C for 15 min. Cells were centrifuged at 120 *g* for 3 min and then fixed using a 50% Carnoy’s solution (acetic acid: methanol ratio, 1:3). The centrifugation and fixing steps were repeated three times. During the last repeat, cells were kept in Carnoy’s solution and dropped onto glass slides. Chromosome spreads were stained with Giemsa solution.

**Teratoma formation.** To generate teratomas, 1–2 × 10^6^ Cm PSCs (CMK6, TRSK, iPS-L3, iPS-L6, iPS-S6, and iPS-S12) before and after naïve-like conversion were injected under the testis of 5–8-week-old severe combined immunodeficiency (SCID) mice. At 4–8 weeks after transplantation, the teratomas were dissected out and fixed in paraformaldehyde. Paraffin wax sections were stained with haematoxylin/eosin, toluidine blue, or alcian blue. To detect neural cells and muscle cells, sections were immunohistochemically analysed using an anti-neural specific enolase antibody and anti-α-smooth muscle actin antibody (Dako, Glostrup, Denmark), respectively. Immunopositive signals were detected using an avidin-biotinylated peroxidase complex method.

**Electron microscopy of naïve-like converted Cm ESCs.** Cm ESC lines, CMK6 and TRSK, before and after naïve-like conversion were fixed in a mixture of 2.5% glutaraldehyde and 2% paraformaldehyde in 0.1 M phosphate buffer (pH 7.4) overnight at 4 °C. The fixed cells were then washed in phosphate buffer and post-fixed in 1% osmium tetroxide in phosphate buffer for 60 min on ice. They were then dehydrated and embedded in Epoxy resin, after which ultrathin sections (60–80 nm thick) were cut, stained with 2% uranyl acetate in 70% methanol and Reynolds’ lead citrate, and observed in a transmission electron microscope (HT-7700; Hitachi, Tokyo, Japan) operating at 80 kV.

**2-Deoxyglucose assay.** Cm ESC lines, CMK6 and TRSK, before and after naïve-like conversion were seeded with the same numbers of cells (4 × 10^4^/well) and cultured on feeder free condition in the absence and in the presence (2 mM and 4 mM) of 2 deoxyglucose (2DG) for 5 days. To prevent apoptosis according to the single dissociation of primed-state ESCs, cells were cultured in the presence of a Rock inhibitor, Y27632, for 2 h before and after passage. Alkaline phosphatase (AP) positive cells were detected and quantified with a BZ-9000 Series All-in-One Fluorescence Microscope and BZII image analysis system (Keyence).

**Oxygen consumption rate (OCR) measurement using Seahorse cellular flux assays**. OCR was measured using an XFp extracellular flux analyser (Seahorse Biosciences, Billerica, MA, USA) using the manufacturer’s protocol. In brief, naïve-like and primed ESCs were seeded onto XFp Seahorse miniplates precoated with laminin 511 at 4 × 10^4^ cells per well and cultured overnight before the experiment. To prevent apoptosis according to the single dissociation of primed-state ESCs, cells were cultured in the presence of a Rock inhibitor, Y27632, for 2 h before and after passage. Culture media were exchanged for XF Base Medium (Seahorse Biosciences) supplemented with 1mM sodium pyruvate and 10 mM glucose with an adjusted pH of 7.4, and cells were incubated at 37 °C under 5% CO_2_ in humidified air for 1 h. Oligomycin (1.0 μM), 4-(trifluoromethoxy)phenylhydrazone (FCCP, 1.0 μM), antimycin (0.5 μM), and rotenone (0.5 μM) were injected during the assay (XF Cell Mito Stress test kits, Seahorse Biosciences). The OCR values were normalized to the number of cells present in each well. Changes in OCR in response to the addition of substrates and inhibitors were defined as maximal changes after their injection compared with the last OCR value measured before the injection.

**Quantitative assessment of neural differentiation.** A quantitative immunocytochemical analysis of the differentiated immunopositive cells was performed after their differentiation was induced from the same numbers of uniformly sized embryoid bodies (EBs; 6 per well) for the same duration. The immunopositive cells were quantified using a BZ-9000 image analyser. Because the core region of the EB attachment area constituted a large immunopositive cluster, it was not counted as a single immunopositive cell. Instead, the immunopositive area per unit area of the well was measured. The area of DAPI-labelled nuclei on each image was defined as the total cell area per unit area of the well. The immunopositive area/DAPI area was used as the “neural differentiation index”. To evaluate the difference of differentiation capacity before and after naïve-like conversion between PSC lines, relative differentiation indices were compared.

# References

1 Thomson, J.A. *et al*. Embryonic stem cell lines derived from human blastocysts. *Science* **282**, 1145-1147 (1998).

2 Hasuwa, *H. et a*l. Transgenic mouse sperm that have green acrosome and red mitochondria allow visualization of sperm and their acrosome reaction in vivo*. Exp An*i**m** 59, 105-107 (2010).
